# All Eyes on Hyperkalemia: A Case Report of Timolol Ophthalmic Drops Leading to Hyperkalemia

**DOI:** 10.1016/j.xkme.2026.101322

**Published:** 2026-03-11

**Authors:** Michael Dwilow, Brie Seniuk, Francis Remulla, Paul Komenda, Jay Hingwala

**Affiliations:** 1 Winnipeg Regional Health Authority Pharmacy Program, Winnipeg, MB, Canada; 2 Department of Food and Human Nutritional Sciences, University of Manitoba, Winnipeg, MB, Canada; 3 Health Sciences Centre, Winnipeg, MB, Canada; 4 Department of Internal Medicine, Max Rady College of Medicine, University of Manitoba, Winnipeg, MB, Canada; 5 Department of Nephrology, Max Rady College of Medicine, University of Manitoba, Winnipeg, MB, Canada; 6 Chronic Disease Innovation Centre, Seven Oaks General Hospital, Winnipeg, MB, Canada

**Keywords:** TK, hyperkalemia, topical ophthalmic

## Abstract

Nonselective β-blockers are a class of medication that competitively antagonize the β_1_ and β_2_ adrenergic receptors. Nonselective β-blockers include such agents as timolol, propranolol, and nadolol, among others. In particular, certain nonselective β-blockers may be used as intraocular pressure-lowering agents for the treatment and management of elevated intraocular pressure and glaucoma. Herein, we present a case of chronically elevated plasma potassium levels in a patient with glaucoma, attributable to the systemic hyperkalemic effects of the ophthalmically administered nonselective β-blocker timolol, an adverse effect which has only rarely been reported. Discontinuation of ophthalmic timolol—and a switch to a different intraocular pressure-lowering agent—resulted in the prompt normalization of plasma potassium levels and reversal of the hyperkalemia, without the need for any further subsequent interventions. The aim of this report is to highlight the potential for topical ophthalmic timolol to cause hyperkalemia, as well as propose potential mitigation and management strategies for patients with elevated potassium levels.

Topical administration of nonselective β-blockers, including timolol, is a common strategy for managing intraocular hypertension.

The potential for cardiac and pulmonary adverse effects arising from the systemic absorption of ophthalmically administered timolol (OAT) has been previously described.^1^^,^^2^ Hyperkalemia is an uncommon adverse effect of OAT, with only a single previous case report in the literature.^3^ Clinicians should be aware of this potential to treat and prevent severe hyperkalemia. To demonstrate this, we describe a case of new onset persistent hyperkalemia following initiation of OAT in a patient with glaucoma.

## Case Report

An 80-year-old woman presented to her family physician with routine bloodwork showing an elevated plasma potassium level of 6.0 mmol/L. She was asymptomatic, with no other complaints. Her past medical history included glaucoma. She took no prescription, over-the-counter, supplemental, herbal, or other medications typically associated with hyperkalemia. She had no history of diabetes mellitus, chronic kidney disease, cerebrovascular disease, hypertension, autoimmune disorders, constipation, or other potentially contributory medical conditions. A dietary review found no significant potassium intake.

Repeat bloodwork results showed hyperkalemia on multiple occasions, ranging between 5.5 and 6.1 mmol/L (normal range, 3.5-5.1 mmol/L). This prompted consultation with a general internal medicine specialist. With a plasma potassium level of 5.9 mmol/L, a 24-hour urine output assessment was performed and showed potassium and sodium excretion of 50 mmol and 46 mmol per day, respectively. Her serum cortisol level was 665 nmol/L (normal range, 130-540 nmol/L). Her plasma aldosterone and renin levels were 7.75 ng/dL and 0.66 ng/dL, respectively. Her plasma aldosterone to renin ratio was normal at 11.7 ng aldosterone/ng renin. To treat her hyperkalemia, hydrochlorothiazide 12.5 mg by mouth once daily was prescribed. She developed dizziness and photosensitivity, prompting discontinuation of hydrochlorothiazide and initiation of sodium polystyrene sulfonate 15 grams by mouth thrice weekly on Mondays, Wednesdays, and Fridays. In the ensuing months, she had multiple visits to local emergency departments for asymptomatic elevated plasma potassium levels greater than or equal to 6.0 mmol/L. During this time, her sodium polystyrene sulfonate prescription was increased to 15 grams by mouth 4 times per week. She was referred to our nephrology clinic for management of refractory hyperkalemia.

A medication review noted that she was using a fixed-dose combination of dorzolamide 2%/timolol 0.5% ophthalmic solution (manufactured by Sandoz Canada, Inc), 1 drop to each eye twice daily, for treatment of glaucoma, as well as latanoprostene bunod 0.024% ophthalmic solution. She was normotensive and had normal cardiac, respiratory, abdominal, and neurological examinations.

A review of her prescription history and bloodwork showed that her hyperkalemia ensued shortly after starting OAT. Nonpharmaceutical causes were not felt to be consistent with her clinical presentation and bloodwork, and the only possible culprit medication identified was timolol because the patient did not have metabolic acidosis (which can occur from dorzolamide). Her ophthalmologist was consulted, who discontinued her dorzolamide/timolol and switched to brinzolamide 1% ophthalmic solution.

One month after OAT stopped, she had a follow-up visit to our nephrology clinic and had been taking sodium polystyrene sulfonate for approximately 3 months. No further changes to her medications had been made, and she was tolerating the change to brinzolamide. A 24-hour urine collection showed a total potassium excretion of 23 mmol/day (normal range: 40-80 mmol/day) and sodium excretion of 109 mmol/day. The urine collection was negative for the presence of protein, and creatinine clearance was 118 mL/min/1.73 m^2^. Plasma biochemistry results included a sodium level of 144 mmol/L, potassium level of 4.0 mmol/L, chloride level of 104 mmol/L, bicarbonate level of 27 mmol/L, and a creatinine level of 51 mmol/L. A complete blood count showed a white blood cell count of 3.9 × 10^9^ cells/L, hemoglobin level of 134 g/L, and a platelet count of 261 × 10^9^ cells/L. Urinalysis showed no abnormalities, and the urine albumin to creatinine ratio was 3.3 mg/mmol. Because her potassium level normalized, sodium polystyrene sulfonate was discontinued. Subsequent follow-up bloodwork at 2, 4, and 12 weeks showed plasma potassium levels of 5.0, 4.2, and 4.0 mmol/L, respectively.

## Discussion

Prompt and definitive management of hyperkalemia is important because it may cause clinical symptoms, including nausea, vomiting, diarrhea, fatigue, muscle cramps, neuromuscular weakness, paresthesia, and flaccid paralysis, or lead to cardiac arrhythmias, cardiac arrest, and sudden cardiac death.^4^^,^^5^

In nonemergent presentations of hyperkalemia, including chronic hyperkalemia, a thorough review of all potential contributory factors should be performed. A dietary review by a registered dietitian with expertise in the management of patients with kidney disease and electrolyte disorders should be conducted to identify and mitigate any potential sources of excess exogenous potassium.^6^ A review of a patient’s entire medication history, including prescription, and nonprescription/over-the-counter medications, as well as herbal products, supplements, and therapies from alternative medical systems (eg, naturopathic, homeopathic, Ayurvedic, and traditional Chinese) should be conducted. This can occur by or in consultation with a pharmacist with training or experience in the management of patients with kidney disease and electrolyte disorders.^6^ Nonessential medications potentially contributing to hyperkalemia should be considered for discontinuation or substitution.^6^ Several classes of medications may be potential causes of iatrogenic hyperkalemia, including β-blockers, calcineurin inhibitors, epithelial sodium channel blockers, heparin, ketoconazole, mineralocorticoid receptor antagonists, nonsteroidal anti-inflammatory drugs, and renin–angiotensin system inhibitors. Pharmacotherapy of chronic hyperkalemia may involve the application of one or more therapeutic agents. In patients with metabolic acidosis, the administration of oral sodium bicarbonate may be considered.^6^ Diuretics may be used to promote excretion of potassium via the kidneys.^6^ Potassium-binding resins may be used to increase excretion of potassium via the gastrointestinal tract.^6^ Algorithms outlining the appropriate management of emergent and nonemergent hyperkalemia have been published previously.^6^

Nonselective β-blockers may exhibit a greater propensity to cause hyperkalemia than cardio-selective β-blockers through greater affinity for the β_2_ receptors over the β_1_ receptors.^7^^,^^8^ Although this interaction does not increase total body potassium levels, it decreases the influx of potassium intracellularly.^7^ Pharmacokinetic studies have shown that plasma timolol concentrations following intravenous infusion resulted in systemic beta blockade, evidenced by significant cardiopulmonary effects (eg, decreased respiration, decreased heart rate).^9^^,^^10^ Plasma timolol concentrations after topical ophthalmic administration are comparable to those produced after intravenous infusions of timolol.^11^^,^^12^ In the study by Korte et al,^11^ subjects exposed to single topical ophthalmic doses of 0.2 mg of timolol experienced systemic cardiopulmonary effects of β blockade. Our patient was using a product containing timolol 5 mg/mL. The dropper provided with this product produces drops ranging in size from 0.031 to 0.115 mL.^7^ This equates to 0.620 to 2.300 mg per day, for patients receiving 4 drops total. This is equivalent to 310% to 1,150% of the dose shown by Korte et al^11^ to cause systemic β blockade.

Although an improper administration technique was not suspected as a significant factor in this case, health care providers should assess all patients receiving topical ophthalmic medications for proper techniques to reduce systemic exposure, especially when these medications may cause systemic adverse effects. Effective techniques for reducing systemic exposure to topically administered ophthalmic medications include nasolacrimal occlusion and eyelid closure.^10^ These are highly effective techniques, reducing systemic exposure to timolol by more than 60%.^10^

Our patient was receiving timolol as part of a fixed-dose combination of OAT alongside 2% dorzolamide, a carbonic anhydrase inhibitor. Although carbonic anhydrase inhibitors may cause metabolic acidosis, potentially resulting in hyperkalemia, our patient’s plasma bicarbonate level was consistently within the normal range. Additionally, on switching to brinzolamide (another carbonic anhydrase inhibitor), her hyperkalemia resolved, reducing suspicion that carbonic anhydrase inhibitors were implicated.

Our patient’s potassium excretion in the 24-hour urine collection was lower than would typically have been expected with hyperkalemia, raising the possibility of an underlying aldosterone deficiency or resistance that was unmasked by the use of timolol, supported by her elevated serum cortisol level of 665 nmol/L. This may represent a normal response to hyperkalemia, and—given the patient’s plasma aldosterone to renin ratio was normal at 11.7 ng aldosterone/ng renin—we felt that a primary mineralocorticoid deficiency was unlikely. Although our patient’s 24-hour urine potassium level decreased following discontinuation of OAT, the subsequent lower urine potassium value may better represent our patient’s “true” baseline, with the earlier greater urine potassium reflecting a compensatory hyperkaliuric response to elevated plasma potassium in the presence of OAT.

Occasionally, there may be significant intrapatient variability in potassium levels because of several factors: variations in circadian patterns of potassium excretion, consumption of potassium-containing substances, collection methods, or analytical techniques (ie, serum vs plasma potassium measures).^6^^,^^13^^,^^14^

Certain cases of hyperkalemia may be spurious—or pseudohyperkalemia—and therefore not a true reflection of patients’ potassium status, including following improper sampling technique, inappropriate storage and transportation, or laboratory error.^13^

Potassium levels may be affected by potassium dietary load, especially in patients with chronic kidney disease.^14^ Although the relevance of such transient elevations in potassium levels is unclear, they are a potential cause of laboratory-identified hyperkalemia in some patients.

Our case highlights the importance of maintaining awareness of the potential for adverse effects following ophthalmic administration of timolol because it is not often considered as a cause of hyperkalemia or other systemic adverse effects.^1^^,^^2^^,^^8^^,^^9^^,^^11^ Hyperkalemia and other systemic adverse effects related to the use of topical ophthalmic medications may be obviated by proper administration technique.
